# Design and Performance Analysis of an Intrinsically Safe Ultrasonic Ranging Sensor

**DOI:** 10.3390/s16060867

**Published:** 2016-06-13

**Authors:** Hongjuan Zhang, Yu Wang, Xu Zhang, Dong Wang, Baoquan Jin

**Affiliations:** Key Laboratory of Advanced Transducers and Intelligent Control Systems, Ministry of Education, Taiyuan University of Technology, No. 79 Yingzexi Street, Taiyuan 030024, China; zhanghongjuan@tyut.edu.cn (H.Z.); wangyu@tyut.edu.cn (Y.W.); zhx15198858616@163.com (X.Z.); wangdongwind@gmail.com (D.W.)

**Keywords:** ultrasonic ranging sensor, driving circuit, impedance matching circuit, intrinsically safe circuit, dual level protection

## Abstract

In flammable or explosive environments, an ultrasonic sensor for distance measurement poses an important engineering safety challenge, because the driving circuit uses an intermediate frequency transformer as an impedance transformation element, in which the produced heat or spark is available for ignition. In this paper, an intrinsically safe ultrasonic ranging sensor is designed and implemented. The waterproof piezoelectric transducer with integrated transceiver is chosen as an energy transducing element. Then a novel transducer driving circuit is designed based on an impedance matching method considering safety spark parameters to replace an intermediate frequency transformer. Then, an energy limiting circuit is developed to achieve dual levels of over-voltage and over-current protection. The detail calculation and evaluation are executed and the electrical characteristics are analyzed to verify the intrinsic safety of the driving circuit. Finally, an experimental platform of the ultrasonic ranging sensor system is constructed, which involves short-circuit protection. Experimental results show that the proposed ultrasonic ranging sensor is excellent in both ranging performance and intrinsic safety.

## 1. Introduction

Non-contact distance measurement is widely used for aircraft altimetry, ship docking, vehicle safety reversing and some other location detection systems. However, non-contact distance measurements in flammable and explosive environments such as coal mines, and the chemical, oil, gas industries and some other circumstances have always been a technical challenge.

In general, there are several mature non-contact distance measurement technologies, such as capacitive [1], laser [2,3], infrared [4,5], and ultrasonic [6,7] methods, *etc*. The capacitive sensor is normally used in near-distance measurements whose range is within 0.3 m [8]. Laser distance measuring sensors show advantages of high accuracy, anti-electromagnetic interference, and their measurement distance can be up to 1000 m. They are often considered suitable for distance measurement in harsh environments. The infrared distance measuring sensor is based on the characteristics of non-proliferation when infrared light is spread. With its advantages of high speed, high resolution and high security, an infrared distance measuring sensor can be used to take a distance measurement of up to hundreds of meters. However, the above two methods have some disadvantages. They both have a small beam-angle, which will lead to a limited direction coverage, and they cannot replace ultrasonic devices in some applications such as an ultrasonic parking assistance systems. The beam-angle of an ultrasonic transducer varies from 7° to 30°, which is a large direction range. The ranging principle of ultrasonic distance measurement systems is based on the time-of-flight measurement of reflected echoes [9,10]. Ultrasonic sensors are increasingly popular compared to other devices in the field of distance measurement up to a few meters, because they have advantages of low cost, easy implementation, anti-electromagnetic interference, and strong ability to adapt all kinds of targets and light intensities [11,12]. For this reason, ultrasonic sensors are widely used in target distance measurement tasks [13,14].

However, in certain flammable and explosive environments, the concentration of flammable gas can be high. It is dangerous to let a distance measuring system to be exposed to this environment, so all the electrical devices need to be installed in explosion-proof shells, which are heavy and limits their use in the field. It is much safer when an ultrasonic ranging sensor is designed with an intrinsically safe circuit. Furthermore, the design makes the intrinsically safe equipment have smaller size and lower cost [15,16]. Intrinsically safe circuits are widely used in flammable and explosive environment applications, such as water level measurement [17] and power supplies [18]. Unfortunately, the applications of currently existing ultrasonic distance measurement systems in potentially flammable or explosive environments are limited due to their non-intrinsic safety. Therefore, it is necessary to design an intrinsically safe ultrasonic ranging system.

Intrinsic safety is a protection method based on the principle of limiting energy in an electrical circuit, which is more favorable than the other existing safety concepts [19,20]. In general, there are two categories of intrinsically safe and associated apparatus, which are “ia” and “ib” respectively. The categories differ in two main aspects: one is the number of specified faults and the other is the value of safety factor [21]. In this paper, the circuit is designed according to the standard of “ia”, which has more stringent requirements. When the system is operating, an intrinsically safe circuit in an electrical apparatus of category “ia” should not cause ignition in each of the following circumstances:

(a) normal operation;
(b) normal operation and with one countable fault;
(c) normal operation and with two countable faults.

Based on the considerations and thorough analyses of the conventional ultrasonic ranging sensor, a novel ultrasonic ranging sensor has been designed and fabricated to achieve intrinsically safe characteristics and to be usable in flammable or explosive environment applications, while maintaining (almost) the same performance and function levels as conventional sensors. Besides the necessary designs to meet the precision and range demands of ultrasonic distance measurement, the intrinsically safe circuit is laid out and evaluated. Moreover, an energy limiting circuit is specially designed to achieve dual over-voltage and over-current protection for the whole sensor system. Finally, a series of experiments are performed to verify the performance of the proposed intrinsically safe ultrasonic ranging sensor.

## 2. An Analysis of Ultrasonic Ranging Sensors and Their Impedance Matching Circuits

**Figure 1:**
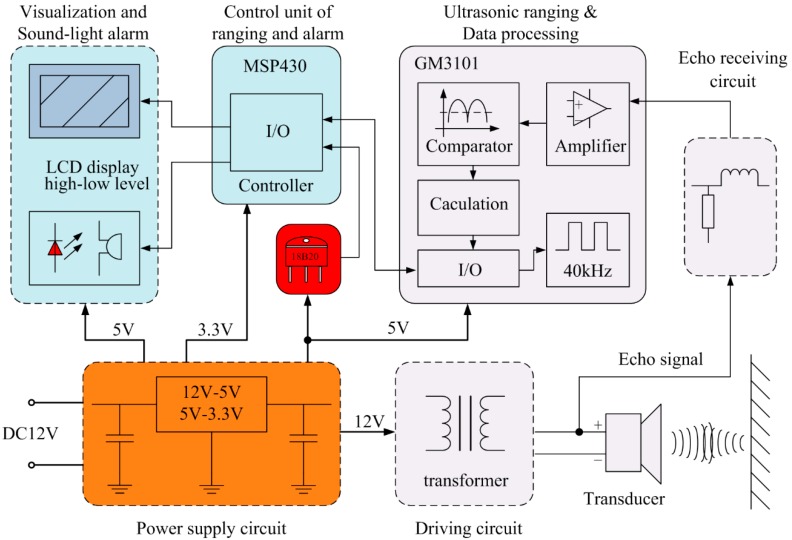
Block diagram of the ultrasonic ranging sensor.

The block diagram of the ultrasonic ranging sensor is illustrated in Figure 1. According to the demands of ultrasonic ranging equipment, such as a reversing ultrasonic alert system, the system should have at least three transducer circuits at the same time due to the directivity characteristics of a single ultrasonic transducer, if not, it is difficult to detect a target outside the beam-angle. In Figure 1, only one transducer circuit is shown. The implementation of the other two is the same as this. As can be easily seen, the whole system is broadly composed of a power supply circuit, driving circuit, transducer, echo receiving circuit, ultrasonic ranging and data processing, DS18B20 temperature compensator (Dallas Semiconductor Inc, Dallas, TX, USA), ranging and alarm control unit, visualization and sound-light alarm.

The power supply circuit provides all kinds of voltage grades for the sensor system. The ranging module is made up of an ultrasonic transmitting circuit, an echo receiving module and data processing module. The GM3101 (Chengdu Goldtel Microelectronics Co., LTD, Chengdu, China) ultrasonic ranging IC (integrated circuit, IC) is in charge of transmitting 40 kHz pulse signals and transforming the echo signals into distance information. The alarm module consists of two parts: one part is the ranging and alarm unit used to send ranging and alarm commands, and the other part is the visualization and sound-light alarm module.

**Figure 2:**
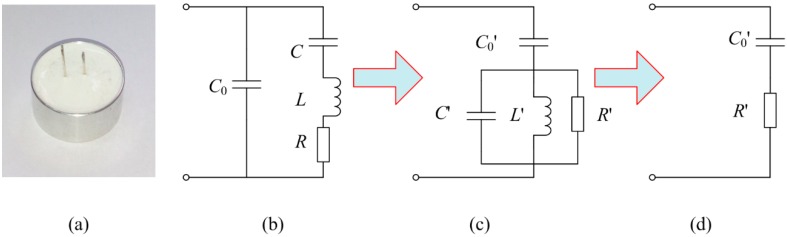
(**a)** The adopted ultrasonic transducer; (**b**) Internal equivalent circuit of ultrasonic transducer; (**c**,**d**) Equivalent circuit transformation when parallel resonant.

In the proposed intrinsically safe ultrasonic ranging sensor, a waterproof piezoelectric transducer with integrated transceiver is selected as the energy transducing element because it is suitable for complex environments, such as coal mines. The appearance and the equivalent circuit diagram of the waterproof piezoelectric transducer with integrated transceiver are schematically shown in Figure 2a,b.

Figure 2a shows the transducer has a metal shell, which can insulate the internal structure from water and dust in the complex environment. Moreover, when the transducer works in resonant state, its equivalent circuit can be depicted as in Figure 2b, where the static capacitance, the dynamic capacitance, the dynamic inductance, and the dynamic resistance of the piezoelectric transducer are defined as *C*_0_, *C*, *L* and *R*, respectively.

When the transducer’s parallel branch is resonant, in other words, working at a parallel resonant angular frequency, the reactance of *L*, *C* and *C*_0_ will be counteracted and the transducer may be equivalent to Figure 2c. *C*′_0_, C′, *L′* and *R′* are respectively the static capacitance, the dynamic capacitance, the dynamic inductance, and the dynamic resistance of the piezoelectric transducer after equivalent transformation. Then, the equivalent circuit can be simplified into Figure 2d through further equivalent transformation [22]. Relevant calculations are expressed as follows:

(1) $$w_{p}=\sqrt{\frac{C+C_{0}}{LCC_{0}}}$$

(2) $$\begin{array}{l} Z_{p}=\frac{1}{\frac{1}{R+jw_{p}L+\frac{1}{jw_{p}C}}+jw_{p}C_{0}}=\frac{1}{\frac{jw_{p}C}{1-w_{p}^{2}LC+jw_{p}CR}+jw_{p}C_{0}}\\ =\frac{1\;w_{p}^{2}LC+jw_{p}CR}{jw_{p}(C+C_{0}-\times w_{p}^{2}LCC_{0})-w_{p}^{2}CC_{0}R}\\ =\frac{1}{w_{p}^{2}C_{0}^{2}R}-j\frac{1}{w_{p}C_{0}} \end{array}$$

where, *w_p_* is the parallel resonance angular frequency [22], and *Z_p_* is the total impedance of the transducer when working at the parallel resonance angular frequency.

After combining Equation (2) and Figure 2d, some conclusions can be drawn as follows:

(3) $$R^{\prime }=\frac{1}{w_{p}^{2}C_{0}^{2}R}$$

(4) $${C^{\prime }}_{0}=C_{0}$$

**Figure 3:**
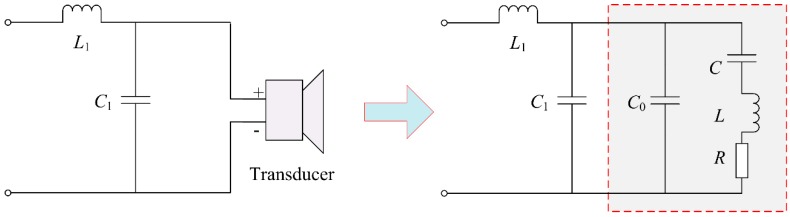
*LC* matching circuit diagram of the piezoelectric transducer.

The piezoelectric transducer is capacitive at resonance. In order to improve the vibration amplitude of the surface and the work efficiency of piezoelectric transducer, the problem of impedance matching must be solved first. Generally, there are various impedance matching circuits such as parallel inductance, series inductance, capacitance-inductance, T network, *etc*. Among them, the capacitance-inductance matching circuit is the most practical, for it can not only match the transducer but also wipe off the harmonic from the power source. As can be seen from Figure 3, *L*_1_ is the matching inductance and *C*_1_ is the matching capacitance.

To simplify the calculations, the total shunt capacitance of both ends of the transducer is expressed as *C_S_* = *C_1_* + *C_0_*. Hence, the total input impedance of piezoelectric transducer can be derived by substituting the *C_S_* expression into Equation (2). The following equations can be obtained:

(5) $${Z^{\prime }}_{p}=\frac{1}{w_{p}^{2}c_{s}^{2}R}+j(w_{p}L_{1}-\frac{1}{w_{p}C_{s}})$$

where, *Z*′_p_ is the total impedance after the impedance matching. Then the imaginary part is set as 0, the relationship between the matching capacitance and the matching inductance is obtained, as is shown below:

(6) $$w_{p}=\frac{1}{\sqrt{L_{1}C_{s}}}=\frac{1}{\sqrt{L_{1}(C_{1}+C_{0})}}$$

Equation (6) shows that the parameters of *L*_1_ and *C*_1_ only depend on *C*_0_, the static capacitor of piezoelectric transducer, when the piezoelectric transducer is working in the state of parallel resonance. Relevant parameters of the selected piezoelectric transducer and matching circuit can be seen in Table 1. Moreover, all of the parameters are obtained under the condition of 48 V driving voltage and are allowed for an intrinsic safety design. The experimental studies will be conducted and analyzed in next section.

## 3. Analysis of Non-Intrinsically Safe Ultrasonic Driving Circuit

**Figure 4:**
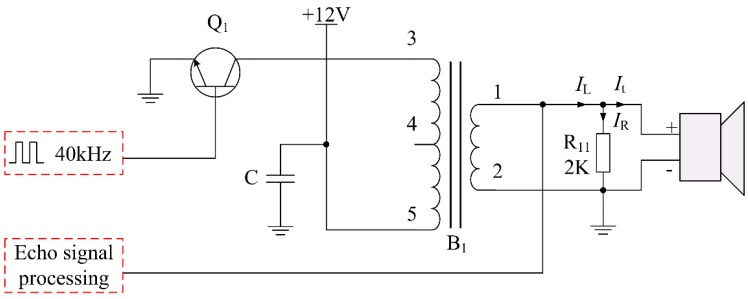
Typical driving circuit of conventional ultrasonic ranging sensor.

Compared to the non-waterproof transducer, the waterproof piezoelectric transducer with an integrated transceiver can be used in harsh environments. However, it has lower transmitting and receiving efficiency [23]. The waterproof piezoelectric transducer with an integrated transceiver cannot be driven directly by a microprocessor. Therefore a specially designed driving circuit is required. For the ultrasonic ranging sensor, a power supply circuit and a driving circuit are the main energy circuits. A typical driving circuit of conventional ultrasonic ranging sensor is shown in Figure 4.

In Figure 4, B_1_ is the special intermediate frequency transformer to provide enough driving voltage (about 48 V) for the ultrasonic transducer. It also has the function of matching the impedance. The inductance coil of B_1_’s high-voltage side and the capacitance of transducer can form a resonant circuit. However, the intermediate frequency transformer has such a large inductance (7.8 ± 0.2 mH) that the driving circuit is difficult to meet the requirement of an intrinsically safe circuit. A specific energy feature analysis is shown below:

(7) $$R^{\prime }=\frac{1}{w_{p}^{2}C_{s}^{2}R}=\frac{1}{{\left(2\times \pi \times 40\times 10^{3}\right)}^{2}\times {\left(23.9\times 10^{-9}\right)}^{2}\times 200}=138.58\;\Omega$$

(8) $$Z_{p}=R^{\prime }-j\frac{1}{w_{p}C_{s}}=(138.58-j166.48)\;\Omega$$

In the stage of ultrasonic wave transmission, the current in each branch of the driving circuit and the current through the inductor are calculated as follows:

(9) $$I_{t}=\frac{U_{0}}{Z_{p}}=(\frac{48}{138.58-j166.48})\text{ A}=(0.141+j0.170)\text{ A}$$

(10) $$I_{R}=\frac{U_{0}}{R_{11}}=(\frac{48}{2\times 1000})\text{ A}=0.024\text{ A}$$

(11) $$I_{L}=I_{t}+I_{R}=(0.165+j0.170)\text{ A}=0.236∠46.0{}^{\circ}\text{ A}$$

where, *I*_L_, *I*_R_, and *I*_t_ are the current of each branch, respectively. *I*_L_ is also the current through the inductor. *U*_0_ is the driving voltage about 48 V. *R*_11_ is the current limiting resistance. In order to improve reliability of the circuit, the current should be multiplied by a safety factor *k* (2.0), which is:

(12) $$I_{L}^{\prime }=k\times I_{L}=(2.0\times 0.236)\text{ A}=0.472\text{ A}$$

According to the critical ignition curve of an inductance circuit [21], we can find the minimum igniting current for 8 mH inductance is *I*_B_ = 300 mA when the voltage is 36 V. Hence, it is believed that the *I*_L_′ is much higher than the minimum igniting current under the condition of 48 V voltage. Therefore, the typical driving circuit has been proved to have non-intrinsically safe features by the calculation of current.

## 4. Analysis of Intrinsically Safe Ultrasonic Driving Circuit

### 4.1. Energy Limiting Circuit

**Figure 5:**
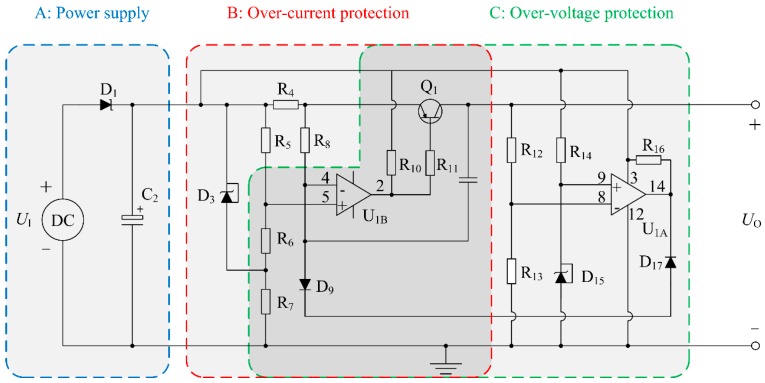
Energy limiting circuit.

In the proposed distance measurement sensor system, an energy limiting circuit is designed to develop the intrinsically safe characteristic of the sensor. The energy limiting circuit is shown in Figure 5.

#### Over-Voltage Protection

As can be seen from Figure 5, the transistor Q_1_ is an on-off component that is controlled by the comparator U_1A_ and U_1B_. In normal state, the base of the transistor Q_1_ is in low level, so the transistor Q_1_ is at an ON-state. D_15_ is TL431, a 3-terminal voltage stabilizer, which can keep *U*_9_ (the voltage of positive input end of U_1A_) at a holding potential of 2.5 V. Namely, *U*_9_ = 2.5 V. Moreover, *U*_8_ (the voltage of inverting input end of U_1A_) can be represented as:

(13) $$U_{8}=\frac{U_{O}\times R_{13}}{R_{12}+R_{13}}$$

**Figure 6:**
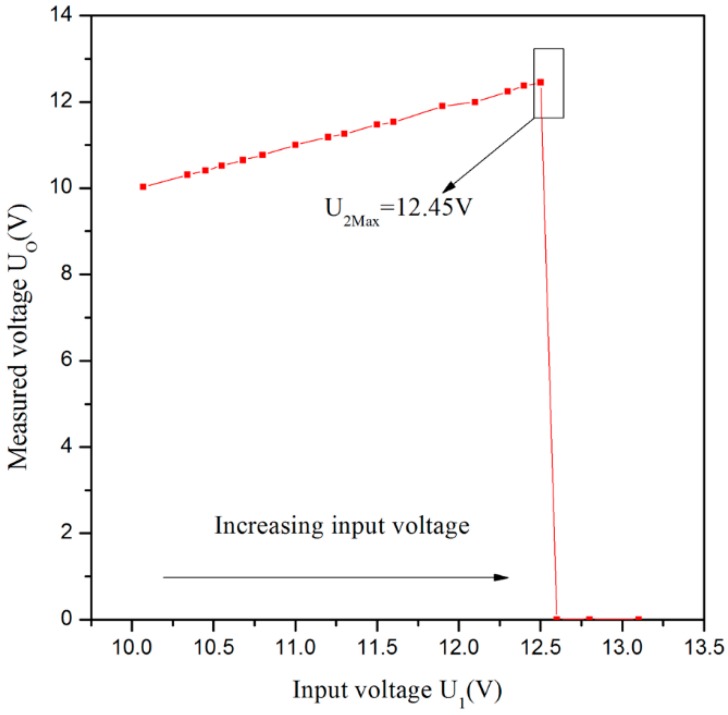
Over-voltage protection curve.

In the proposed sensor system, the threshold of input voltage *U*_I_ is 12.5 V. After matching the suitable value of R_12_ with R_13_, the divider resistors *U*_8_ will achieve 2.5 V when the input voltage *U*_I_ is 12.5 V. If the input voltage is higher than 12.5 V, it will lead to the result that *U*_8_ is greater than *U*_9_, and that the output voltage level of the comparator U_1A_ will change from high to low. Hence, the inverting input end voltage of U_1B_ will be pulled down through the circuits of D_9_ and D_17_, which will cause the base of Q_1_ turning to a high voltage. Then, the transistor Q_1_ is at an OFF-state, in which the power supply will be cut off to realize an over-voltage protection. From Figure 6, we can know that the output voltage *U*_O_ will be 0 V when the input voltage *U*_I_ is higher than 12.45 V.

In the proposed sensor, the threshold current of system is 100 mA. R_4_, whose value is 2 Ω, is the resistance for over-current detecting. Moreover, because D_3_ is an accurate voltage stabilizer TL431 (Texas Instruments, Dallas, TX, USA), the voltage between cathode and anode of D_3_ is 2.5 V. Hence, the positive input end voltage of U_1B_ can be expressed as:

(14) $$U_{5}=U_{I}-U_{R5}=U_{I}-\frac{2.5\times R_{5}}{R_{5}+R_{6}}$$

where *R*_5_ and *R*_6_ are the divider resistors, respectively, *U*_I_ is the input voltage, and *U*_R5_ is the voltage of *R*_5_. As can be seen from Equation (14), by matching the values of *R*_5_ with *R*_6_, the voltage of *U*_R5_ is stabilized at 0.2 V.

**Figure 7:**
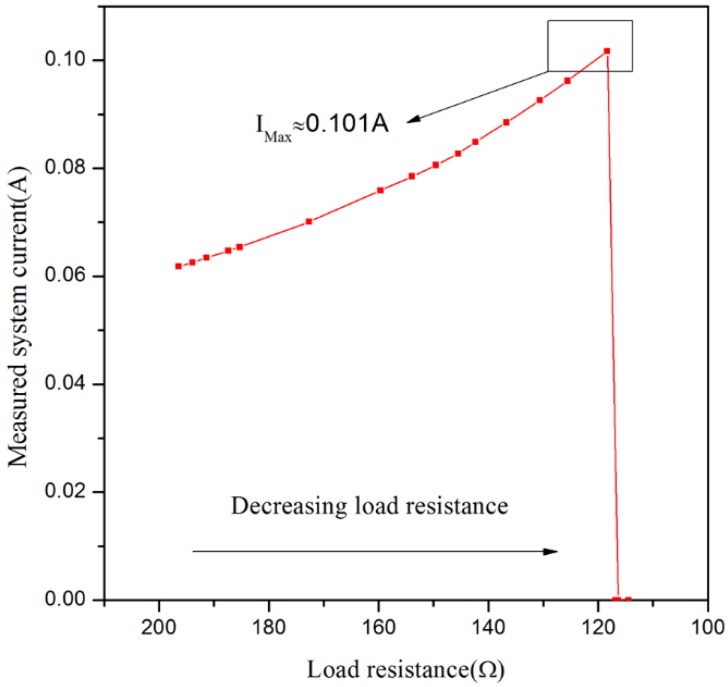
Over-current protection curve.

As the rated current of the proposed sensor is about 70 mA, the voltage of *R*_4_ is lower than 0.2 V under normal circumstances, so for the comparator U_1B_ (LM339, Texas Instruments, Dallas, TX, USA), the voltage of its positive input end is lower than that of inverting input end, which will make the output voltage of U_1B_ become low and Q_1_ be at an ON-state, but when short circuits and other faults occur, the system current is more than 100 mA. Furthermore, the voltage of *R*_4_ will be higher than 0.2 V, which will lead to the output becoming high and Q1 being at an OFF-state. Then, the power supply will be cut off so as to realize over-current protection. Figure 7 shows the maximum allowable current of sensor is 0.101 A.

### 4.2. Design and Evaluation of an Intrinsically Safe Driving Circuit

**Figure 8:**
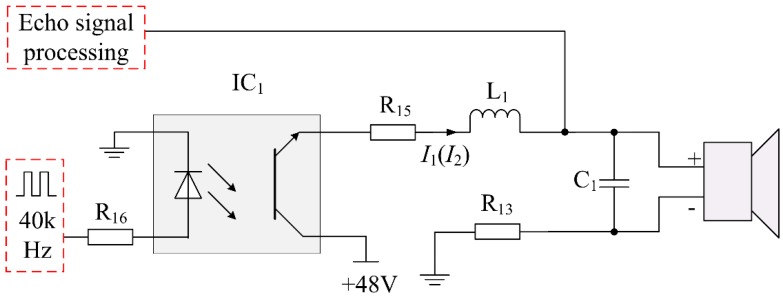
Intrinsically safe driving circuit with high-speed opto-coupler.

To solve the above issue, a transducer driving circuit with a high-speed opto-coupler has been designed. The proposed driving circuit is shown in Figure 8, where IC_1_ is the high-speed opto-coupler, and its switching speed can reach microsecond grade. Once the high speed opto-coupler closes, it can isolate the power supply from an echo signal completely. Therefore it is suitable for transmitting impulse signal and weak echo signal. At the same time, there is no energy storage element in the proposed driving circuit. It can be concluded that the proposed driving circuit meets the requirement of an intrinsically safe circuit based on the above calculation and analysis. The sparks safety assessment of transducer’s matching circuit and drive circuit can be seen as follows:

(15) $$R^{\prime }=\frac{1}{w_{p}^{2}C_{s}^{2}R}=\frac{1}{{\left(2\times \pi \times 40\times 10^{3}\right)}^{2}\times {\left(23.9\times 10^{-9}\right)}^{2}\times 200}=138.58\;\Omega$$

First, under normal condition, the normal work current *I*_1_ of the designed driving circuit is expressed as:

(16) $$I_{1}=\frac{U_{0}}{R_{15}+R_{13}+R^{\prime }}=(\frac{48}{135+135+138.58})\text{ mA}=117.48\text{ mA}$$

where *U*_0_ is the driving voltage about 48 V. The current limiting resistances *R*_13_ and *R*_15_ are both 135 Ω. *I*_1_ is the current of flowing through the inductor *L*_1_. In order to improve reliability of the circuit, the current should be multiplied by a safety factor *k* (2.0), that is:

(17) $$I_{1}^{\prime }=k\times I_{1}=(2.0\times 117.48)\text{ mA}=234.96\text{ mA}$$

According to the critical ignition curve of inductance circuit [21], we can find the minimum igniting current *I*_B_ of 0.33 mH inductance is 1.1 A, which is much higher than 234.96 mA. So, the designed circuit meets the requirements of the desired intrinsic safety circuit.

Second, when the short circuit occurs in the transducer, the current *I*_2_ of the proposed driving circuit is calculated as follows:

(18) $$I_{2}=\frac{U_{0}}{R_{13}+R_{15}}=(\frac{48}{135+135})\text{ mA}=177.78\text{ mA}$$

where *I*_2_ is the shorted current of *L*_1_. If the current *I*_2_ is multiplied by a safety factor *k* (2), that is:

(19) $$I_{2}^{\prime }=k\times I_{2}=(2.0\times 177.78)\text{ mA}=355.56\text{ mA}$$

As it can obviously seen, $I_{B}\gg I_{2}^{\prime }$, which meets the requirements of the desired intrinsic safety circuit.

If the current *I*_2_ is multiplied by a safety factor *k* (1.5), that is:

(20) $$I_{2}^{\prime \prime }=k\times I_{2}=(1.5\times 177.78)\text{ mA}=266.67\text{ mA}$$

According to the critical ignition curve of resistance circuit [21], we can find the minimum igniting current *I*_B_ corresponding to the 48 V power supply is 275.00 mA, which is higher than 266.67 mA. Therefore, the above spark ignition analyses and calculation results indicate that the circuit is intrinsically safe.

**Figure 9:**
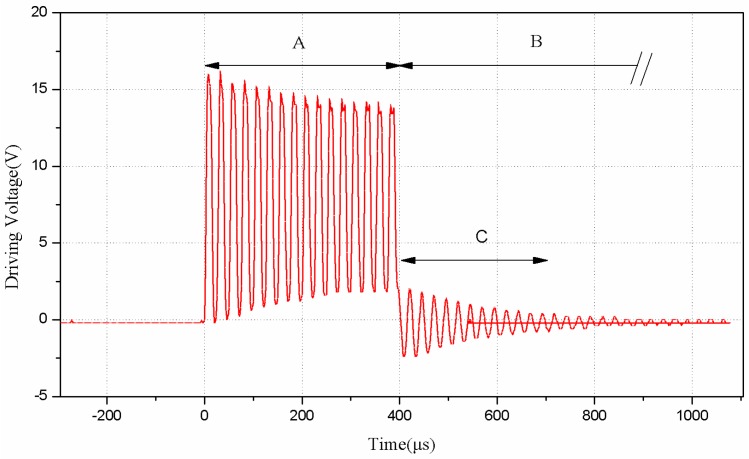
The measured drive pulse of transducer.

**Figure 10:**
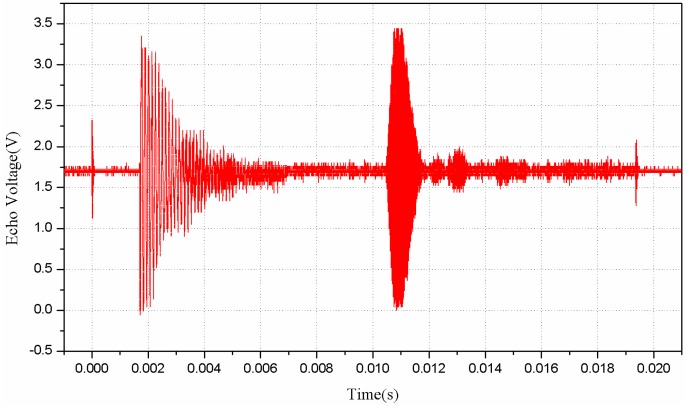
The measured echo voltage of transducer.

The transmitting pulses produced by the novel approach of driving current are shown in Figure 9. Due to the resistance distribution of current limiting resistor and opto-coupler, a pulse string, composed of about 16 V and 40 kHz pulse signals which is controlled by GM3101, will be sent to the anode of the transducer. The time of the stage A, when the sensor is transmitting pulses, is *T*_A_ = 16 × 25 μs = 400 μs. After transmitting the pulses, the microprocessor will turn off the opto-coupler and the transducer will go to the stage of aftershock (C) before the echo receiving stage. In order to distinguish the aftershock signal and the echo signal, a period of aftershock shielding time is necessary, after which the proposed sensor begins to detect an echo. In Figure 9, B represents the stage of aftershock shielding. Obviously, *T*_B_ is much larger than *T*_C_. After designing and implementing the driving circuit, we take a waterproof piezoelectric transducer with an integrated transceiver for experiments to test its driving ability. Tests have been done in various distances of the target object, and the echo waveform in 185 cm is shown in Figure 10. We can find that the echo voltage is still very large (about 1.75 V) in such a long distance, which shows that the proposed novel driving circuit has a pretty good performance in driving the waterproof piezoelectric transducer with integrated transceiver.

## 5. Experimental Demonstration and Discussion

### 5.1. The Realization of the Distance Measurement Sensor

The digital circuit hardware selection is shown in Table 2.

**Figure 11:**
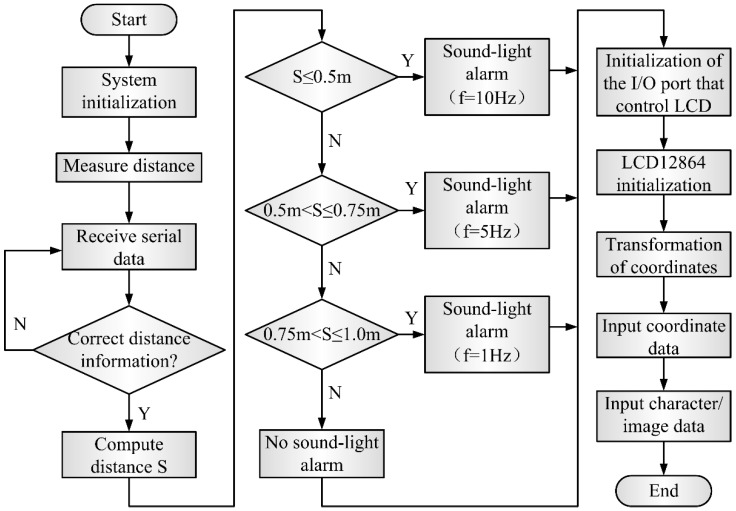
Flowchart of the software.

A software program for the MSP430F149 microprocessor (Texas Instruments, Dallas, TX, USA) has been developed. The flowchart of the software is given in Figure 11. The program was written in C language on the platform of Keil uVision 4 IDE (Advanced RISC Machines, Cambridge, UK). After the system was initialized, the microprocessor sent the command of measuring distance to GM3101 first and then was ready to receive the distance information from GM3101. For the information from GM3101, the microprocessor determines whether it is correct distance information. If the information is right, it will be analyzed and calculated so that we can get the accurate distance S of the obstacle.

Next, the distance S is compared with the setting thresholds in order to decide whether it is necessary to trigger the alarm. In the proposed system, there are three levels of alarm. When S ≤ 0.5 m, the LED will be lit and the buzzer will beep at the frequency 10 Hz. When 0.5 m < S ≤ 0.75 m, the LED will be lit and the buzzer will beep at the frequency 5 Hz. When 0.75 m < S ≤ 1 m, the LED will be lit and the buzzer will beep at the frequency 2 Hz. When S >1 m, the sound-light alarm will not be triggered. Moreover, the distance information will be displayed on the LCD12864. As can be seen in the Figure 11, after initializing the I/O ports and LCD12864, the microprocessor will transform the coordinates where the characters and images display on the LCD screen into the corresponding coordinates of the LCD screen inside, and then feeds the coordinates into LCD screen through parallel I/O ports. Finally, the microprocessor sends the characters and images that express obstacle distances to LCD still through parallel I/O ports.

### 5.2. Experimental Setup and System Errors

**Figure 12:**
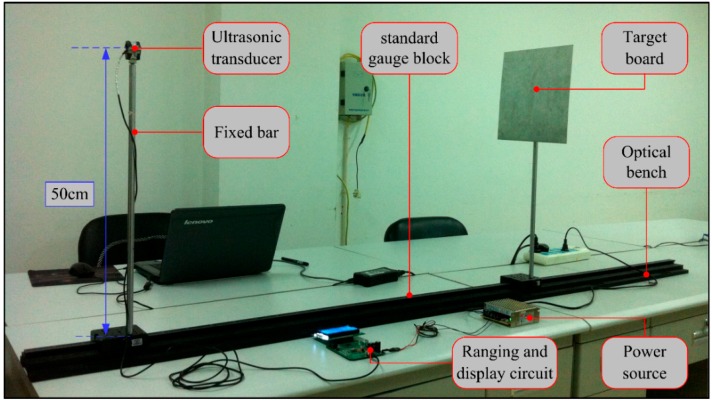
Experimental setup.

To test and verify the effectiveness of the novel transducer circuit and the practicality of the real-time measurement sensor, an experimental measurement has been performed with the intrinsically safe ultrasonic ranging sensor. The entire experimental setup is shown in Figure 12.

An ultrasonic transducer and target object were placed on an optical bench (300 cm in length) with an easily readable distance scale so that the actual distance value was controlled and read precisely by a simple way. In order to avoid the effect of other objects, the transducer and target objects simulating explosion-proof enclosure (25 cm × 25 cm) were fixed at an altitude of 50 cm by two metal rods. The 12 V switching power source provided basic electrical power for the proposed distance measurement sensor. Three types of measurements were performed.

**Figure 13:**
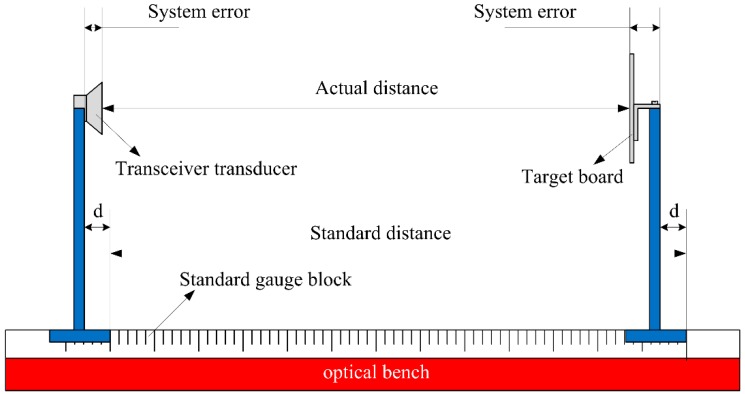
The schematic diagram of the experiment platform (side view).

The schematic diagram of the experiment platform (side view) is shown in Figure 13. From Figure 13, it can be observed that the value read from the standard gauge block is not the actual distance between ultrasonic transducer and target board, due to the errors of test platform. In fact, there is a system error between them, and the actual distance can be expressed as follows:

(21) $$D_{a}=D_{s}-D_{e}$$

where *D*_a_ is the actual distance between transceiver and target board, *D*_s_ is the standard distance that read from the standard gauge block, *D*_e_ is the system error. In these experiments *D*_e_ is 5 cm.

### 5.3. Resolution and Blind Area

For the proposed intrinsically safe ultrasonic ranging sensor, the resolution is mainly determined by the GM3101 distance measurement chip. The details of the GM3101 can be found in the GM3101 datasheet, which indicates a resolution of 5 cm. Although there are higher resolution ultrasonic ranging sensors in existing products, the resolution is acceptable in the practical application considering the purpose of the developed equipment.

A test has been done in order to evaluate the different measuring performance between the proposed intrinsically safe ultrasonic ranging sensor and conventional ultrasonic ranging sensor. We chose several sample points and measured the distance of every sample points by using two categories of ranging sensor respectively. The measured results show that the blind is 30 cm and the maximum measuring range is 300 cm for the proposed intrinsically safe sensor. Within the measurement range of 30 cm to 300 cm, the same measurement results were obtained by adopting two categories of ranging sensor respectively.

### 5.4. Linearity

**Figure 14:**
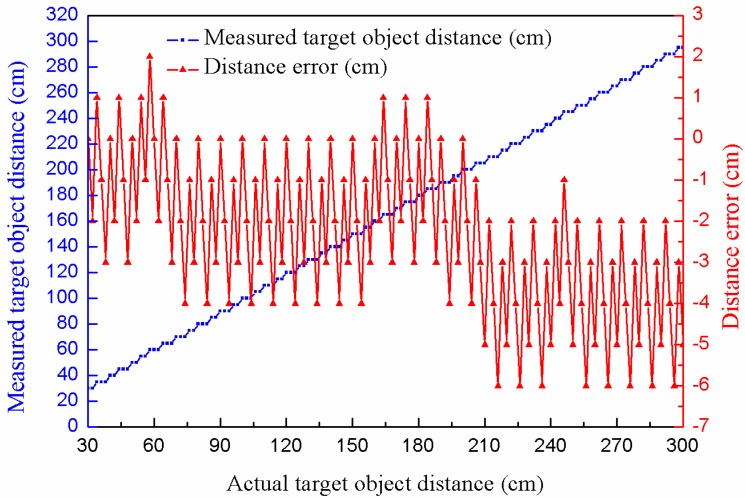
Experimental results of linearity in a range of 300 cm.

Linearity is used to evaluate the performance of the distance measurement sensor. In the experiment, the target object distance is decided by a 300 cm long optical bench, whose precision is 1 mm. Each time, the distance measured by the proposed sensor will be compared with the distance measured by optical bench. According to the interpretation shown in Section 4, we know that the sensor has a period of time for aftershock shielding (*T*_B_). In this paper, the ultrasonic transducer NU40A25TR-1 (Shenzhen JINCI Technology CO., LTD., Shenzhen, China) has been used, consider of the characteristics of it, *T*_B_ was set as 1.7 ms. Hence, the measuring blind area can be obtained as follows:

(22) $$D_{B}=\frac{C\times T_{B}}{2}=(\frac{340\times 1.7}{2\times 1000})\text{ m}=0.289\text{ m}$$

where *D*_B_ is the distance of measuring blind area, *C* is the speed of sound and *T*_B_ is the aftershock shielding time. Experimental results of the linearity test in a measuring range of 30 cm to 300 cm are shown in Figure 14. In Figure 14, it can be seen that the maximum error of this test is about −6 cm or 2.22% within the full scale span (FSS).

The following conclusions can be drawn from the above. Although the sensor is not able to display the merits of the high precision and a wide measuring range, it is able to satisfy the requirement of distance measurement in practical applications. Moreover, the most important advantage is that the proposed sensor is intrinsically safe.

### 5.5. Repeatability

In the experiment, the repeatability of the proposed sensor was also tested specially. Repeatability is the variation of measured value when a surveyor repeatedly measuring the identical characteristic on the same part using a specific measuring instrument. The relative error of repeatability is used for reliability evaluation of measurement sensor. For the proposed sensor, the relative error of repeatability can be expressed as:

(23) $$E_{i}=\frac{L_{\mathrm{iMax}}-L_{\mathrm{iMin}}}{L_{i}}$$

where *E*_i_ is the relative error of repeatability of the sensor, *L*_iMax_ is the maximum measured value of the *i*-th sample points, *L*_iMin_ is the minimum measured value of the *i*-th sample points, and *L_i_* is the standard value of the *i*-th sample points.

**Figure 15:**
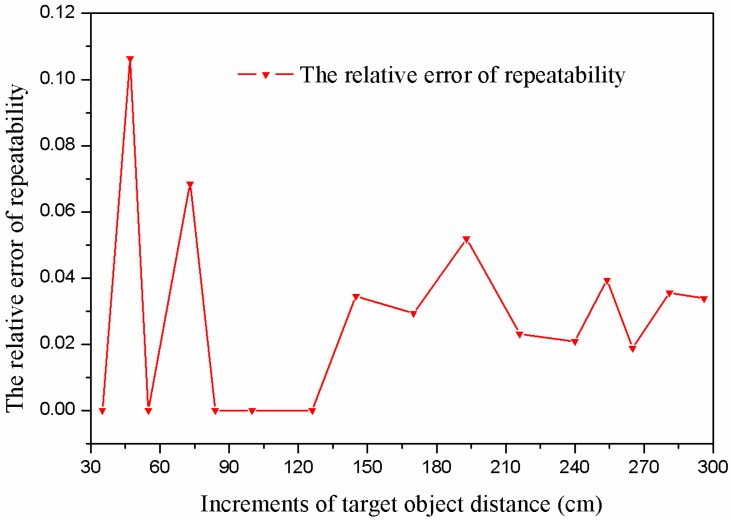
Experimental result of the repeatability test.

In the experiment, at each fixed target object distance, ten measurements are carried out repeatedly. It is observed that there is a small variation among the measurement results. After the measured data are processed, the relative error of repeatability of each sample point can be obtained and is shown in Figure 15, respectively.

As shown in Figure 15, we have selected 15 sample points and carried out 100 measurements in the repeatability experiment. With the increase of the measured distance, in general, the relative error of repeatability of the sensor decreases gradually. The maximum repeatability error is 5 cm or 10.64% of actual value, when the actual value is 47 cm. The minimum repeatability error is 0 cm at several sample points. The relative error of repeatability at close range is seemingly relatively high, because the resolution of the proposed sensor is 5 cm. Moreover, it is necessary to mention that the maximum absolute error of repeatability is 5 cm in a long distance (from 30 cm to about 190 cm). In the range of 190 cm to 300 cm, the maximum absolute error of repeatability is 10 cm. Therefore, for such a wide measurement range, this repeatability of the proposed ranging sensor is excellent.

### 5.6. Short-Circuit Test

**Figure 16:**
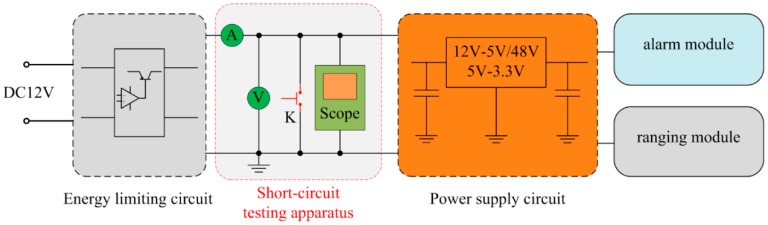
Short-circuit testing of the proposed intrinsically safe ultrasonic ranging sensor.

In order to verify the intrinsically safe feature of the proposed ranging sensor, we have carried out a short-circuit test. The output terminal of the energy limiting circuit is selected as the short-circuit point because the power source short circuit is the worst case. The test scheme is shown in Figure 16.

**Figure 17:**
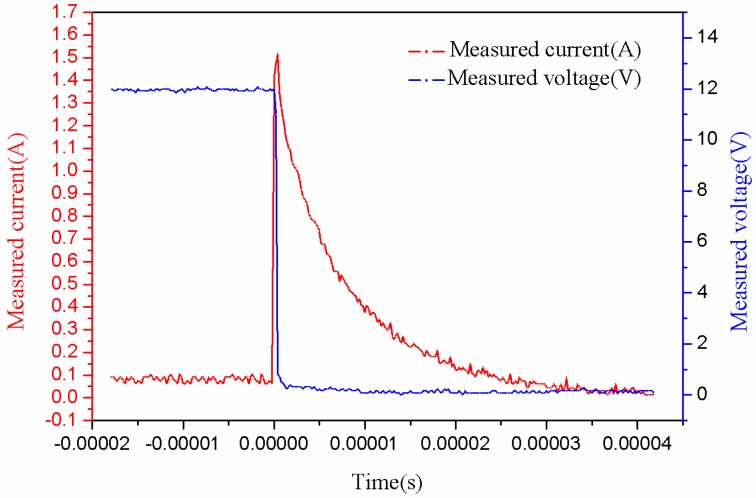
Voltage and current curves in the short-circuit protection testing.

In Figure 16, the part indicated with red font is the schematic diagram of the short-circuit testing apparatus. K is a button switch. The sensor works normally when K is disconnected. However, the output terminal of the energy limiting circuit will be shortened when K is pressed down. The voltmeter and ammeter in testing apparatus are used to measure the voltage and current of the circuit. An oscilloscope is utilized to observe the state of voltage and current in the short-circuit process. The measured results are presented in Figure 17, respectively.

The transient responses of the short-circuit voltage and current are presented in Figure 17. As can be seen, the operating current was about 70 mA and the voltage was about 12 V under the normal state. At the time of 0 s, when the button switch S was pressed down, the measured voltage decreased to about 0 V within 0.4 μs. Furthermore, the current of sensor system surged to 1.5 A within 0.4 μs, and then reduced to 0 A quickly (within 35 μs). Consequently, the test proves that the proposed sensor system can be protected under short-circuit condition. In conclusion, the performance of the proposed intrinsically safe ultrasonic ranging sensor is summarized in Table 3.

## 6. Conclusions

In this paper, an intrinsically safe ultrasonic ranging sensor was successfully designed and demonstrated according to the design requirements of intrinsically safe equipment. The design of the proposed sensor was a comprehensive work of digital circuit design, analog circuit design, and software compilation. The hardware and software of the sensor are developed, with which an intrinsically safe ultrasonic ranging sensor is fabricated. Additionally, as the key energy circuit of sensor, the driving circuit of the transducer is the mainly research object. The analysis of the existing driving circuits of transducers showed with a conventional driving circuit it is difficult to meet the intrinsic safety requirements due to the existence of a large energy storage inductor. Finally, a novel driving circuit approach based on a high-speed opto-coupler was designed. Moreover, its intrinsically safe characteristics were analyzed through detailed derivation and calculation. The calculation results show that the circuit is intrinsically safe. In order to improve the reliability of the sensor system, the energy limiting circuit is designed to provide dual over-voltage and over-current protection for the whole sensor so that the circuit will be protected immediately if the current or voltage exceed a critical value. Through the verification experiments in three aspects: over-voltage, over-current and short-circuit, the critical voltage and critical current are 12.45 V and 0.101 A respectively, and the short-circuit response time is 0.4 μs.

Compared to the traditional ultrasonic ranging sensor, it can be concluded from the experiments that the proposed ultrasonic ranging sensor has good performance and function, and it has an innovative intrinsically safe design. Therefore the proposed sensor is suitable for distance measurements and can be applied in complex hazardous environments such as coal mines, chemical, oil, gas plants and other circumstances. Accordingly, this proposed sensor extends the application of ultrasonic ranging sensors and provides a high security level with easy implementation. An improved version will be further developed to provide simpler circuits, better accuracy, and a smaller blind area.

## Figures and Tables

**Table 1:** Relevant parameters.

Parameter	Value	Uint
*R*	200.00	Ω
*L*	166.64	mH
*L*_1_	0.66	mH
*C*	0.10	nF
*C*_0_	1.90	nF
*C*_1_	22.0	nF

**Table 2:** The digital circuit hardware selection.

Number	Designation	Version	Function
1	Distance measurement chip	GM3101	send and receive ultrasonic signals calculate and send the distance information
2	Microprocessor	MSP430F149	control the work of ranging sensor and receive its measurement results control the work of LCD and sound and light alarm device
3	Display	LCD12864	display the distance information of target object
4	Sound and light alarm	Active buzzer and LED light-emitting diode	Emit a shining light signals Sound alarm

**Table 3:** Performance of the proposed sensor.

Specification	Value	Unit
Resolution	5	cm
Measuring range	300	cm
Maximum nonlinearity error	2.22	% FSS
Maximum repeatability error	10	cm
Critical value of over-voltage protection	12.45	V
Critical value of over-current protection	0.101	A
Short-circuit response time	0.4	μs
